# The influence of different cellular environments on PET radioligand binding: An application to D_2/3_-dopamine receptor imaging

**DOI:** 10.1016/j.neuropharm.2014.05.040

**Published:** 2014-10

**Authors:** Darren R. Quelch, Sarah L. Withey, David J. Nutt, Robin J. Tyacke, Christine A. Parker

**Affiliations:** a Centre for Neuropsychopharmacology, Division of Brain Sciences, Imperial College London, UK; b School of Physiology and Pharmacology, University of Bristol, Bristol BS8 1TD, UK; c Global Imaging Unit, GlaxoSmithKline, UK

**Keywords:** D_2/3_ receptor, PET, Internalisation, [^3^H]PhNO, [^3^H]Raclopride, [^3^H]Spiperone

## Abstract

Various D_2/3_ receptor PET radioligands are sensitive to endogenous dopamine release *in vivo*. The Occupancy Model is generally used to interpret changes in binding observed in *in vivo* competition binding studies; an Internalisation Hypothesis may also contribute to these changes in signal. Extension of *in vivo* competition imaging to other receptor systems has been relatively unsuccessful. A greater understanding of the cellular processes underlying signal changes following endogenous neurotransmitter release may help translate this imaging paradigm to other receptor systems. To investigate the Internalisation Hypothesis we assessed the effects of different cellular environments, representative of those experienced by a receptor following agonist-induced internalisation, on the binding of three D_2/3_ PET ligands with previously reported sensitivities to endogenous dopamine *in vivo*, namely [^3^H]spiperone, [^3^H]raclopride and [^3^H]PhNO. Furthermore, we determined the contribution of each cellular compartment to total striatal binding for these D_2/3_ ligands. These studies suggest that sensitivity to endogenous dopamine release *in vivo* is related to a decrease in affinity in the endosomal environment compared with those found at the cell surface. In agreement with these findings we also demonstrate that ∼25% of total striatal binding for [^3^H]spiperone originates from sub-cellular, microsomal receptors, whereas for [^3^H]raclopride and [^3^H]PhNO, this fraction is lower, representing ∼14% and 17%, respectively. This pharmacological approach is fully translatable to other receptor systems. Assessment of affinity shifts in different cellular compartments may play a crucial role for understanding if a radioligand is sensitive to endogenous release *in vivo*, for not just the D_2/3_, but other receptor systems.

## Highlights

• The internalisation hypothesis was investigated in relation to D_2/3_ receptor PET ligand binding.
• K_*D*_ and B_*max*_ were determined for [^3^H]Raclopride, PhNO and Spiperone in different cellular buffers.
• The cellular distribution of [^3^H]Raclopride, PhNO and Spiperone binding was also determined.
• Reductions in K_*D*_ were observed in the endosomal condition in the following order PhNO > Raclopride > Spiperone.
• K_*D*_ shifts in different cellular compartments may predict sensitivity to neurotransmitter release *in vivo*.

## 1 Introduction

Positron emission tomography (PET) and single photon emission computerised tomography (SPECT) imaging studies are frequently conducted in order to non-invasively investigate regulation of dopamine D_2/3_ receptors (D_2/3_) in healthy and diseased populations (Abi-Dargham, 2004 Volkow et al., 2007). As such, D_2/3_ receptor PET and SPECT studies have contributed vastly to our understanding of dopaminergic neurotransmission in stimulant misuse and schizophrenia (Laruelle, 1998; Abi-Dargham, 2004; Volkow et al., 2007; Martinez and Narendran, 2010). A variety of D_2/3_ radioligands are sensitive to acute fluctuations in dopamine levels *in vivo* (Laruelle, 2000), where the Occupancy Model is generally used to interpret the changes in binding observed. However, temporal discrepancies between the dopamine surge following treatment with compounds such as amphetamine and the change in D_2/3_ PET signal have been identified (Laruelle et al., 1997; Cardenas et al., 2004; Houston et al., 2004; Narendran et al., 2007; Skinbjerg et al., 2010). Furthermore, translation of endogenous release paradigms based on The Occupancy Model to other neurotransmitter systems have been relatively unsuccessful, suggesting that direct competition of radioligand by endogenous neurotransmitter molecule might not sufficiently describe the data.

Agonist-induced internalisation is a cellular mechanism which regulates secondary messenger signalling and functional receptor density at the plasma membrane (Tsao et al., 2001). Typically, following receptor activation and phosphorylation of intracellular domains by G-protein receptor kinases (GRK), the receptor-ligand complex is trafficked towards the endocytic machinery either for lysosomal degradation or to be prepared for re-insertion into the cell membrane. During this trafficking the receptor-ligand complex is exposed to a variety of ionic conditions, depending on its stage in the internalisation pathway. Compared with the D_1_ receptor (Dumartin et al., 1998; Martin-Negrier et al., 2006; Kong et al., 2011), D_2/3_ receptor internalisation is less extensively characterised. However D_2/3_ receptors have been shown to be present in the microsomal and cytosolic compartments in the absence of pharmacological stimulation using immunological based techniques (Paspalas et al., 2006) and a few groups have demonstrated agonist-induced internalisation of the D_2_ receptor in cell and tissue preparations (Kim et al., 2001; Macey et al., 2004; Paspalas et al., 2006). D_3_ receptor internalisation has also been observed following dopamine stimulation in cell systems (Kim et al., 2001; Cho et al., 2007).

An agonist-induced receptor internalisation model has been proposed to contribute to the signal changes observed in endogenous competition PET studies, known as the Internalisation Hypothesis (Laruelle, 2000). Changes in receptor availability or affinity for a radioligand following internalisation may alter observed Binding Potential (BP); since BP is proportional to B_*max*_/K_*D*_. Following this proposal by Laruelle (2000), a number of reports have been made in support of this Internalisation Hypothesis. Of note, Skinbjerg et al. (2010) used an internalisation deficient mouse model, and demonstrated the binding profiles of both [^11^C]MNPA and [^18^F]fallypride better matched the time course of dopamine release following amphetamine in the absence of internalisation (Skinbjerg et al., 2010). Additionally, Guo et al. (2010) have shown the affinity of various D_2/3_ ligands to be altered following quinpirole-induced internalisation in cell systems (Guo et al., 2010). More recently we have reported the ability of the selective serotonin transporter protein (SERT) radioligand, [^3^H]DASB, to bind to the SERT to be significantly reduced in sub-cellular conditions compared with membrane bound conditions utilising an *in vitro* model (Quelch et al., 2012).

The effect of changing sodium concentration and pH on dopamine receptor radioligand binding has been previously reported (Hamblin and Creese, 1982; Sibley and Creese, 1983; Watanabe et al., 1985; Neve, 1991; Sokoloff et al., 1992; Malmberg and Mohell, 1995). However, to our knowledge, the effects of other ions which also differ throughout the agonist-mediated internalisation pathway, on the binding parameters of widely used PET radioligands, have not been investigated as extensively.

In general, the signal associated with any central nervous system PET radioligand is assumed to originate mainly from membrane bound target proteins. However, the contribution to the overall PET signal via the protein target of interest in sub-cellular compartments have not been reported with D_2/3_ receptor PET radioligands. Therefore, any involvement of the target protein in these sub-cellular compartments to the overall observed total PET signal will greatly increase our understanding of the cellular mechanisms involved in endogenous release studies for particular radioligands.

The data presented in this manuscript therefore sought to determine (1) how the agonist induced internalisation pathway may affect the binding parameters of the D_2/3_ radioligands, [^3^H]raclopride, [^3^H]PhNO and [^3^H]spiperone in tissue preparations using *in vitro* methods previously published for other PET radioligands (Quelch et al., 2012), and (2) the full cellular composition of the striatal PET signal for the D_2/3_ radioligands, spiperone, PhNO and raclopride at baseline using an *in vitro* cell fractionation system.

## 2 Methods

### 2.1 Membrane preparation

#### 2.1.1 Rat tissue

Male Sprague Dawley rats (∼250 g) were used for dissection and isolation of striatal tissue. Wet tissue was weighed and homogenised in 10× weight/volume (w/v) Sucrose buffer (0.32 mM Sucrose, 5 mM Tris HCl, 1 mM MgCl_2_ pH 7.4, 4 °C) and centrifuged at 32,000 *g* (20 min, 4 °C). The supernatant was removed and the pellet washed twice by centrifugation (32,000 *g*, 20 min, 4 °C) in Tris buffer (50 mM Tris-Base, 1 mM MgCl_2_, pH7.4, 4 °C). The final pellets were re-suspended in Tris buffer to approximately 10 mg/ml.

#### 2.1.2 Sub-cellular fractionation

The sub-cellular fractionation procedure was based on methods previously presented (Laduron, 1977; Sun et al., 2003). The constituents of the isolated fractions are: P1 = nuclear and cell debris fraction. P2 = plasma membrane and mitochondrial pellet. P3 = microsomal pellet diluted in endosomal buffer and S3 = cytosolic fraction.

The translation from rat to pig tissue was made based on data presented in Supplementary Table 1, Supplementary Fig. 1. Rat and pig D_2/3_ radioligand receptor binding parameters were shown to be altered in a similar manor following exposure to the different cellular environments investigated in this manuscript i.e. lower *in vitro* BP values in the endosomal environment compared with the extracellular, driven by reductions in affinity. Furthermore, use of pig striatum allowed for reductions in animals numbers.

Striatal tissue (3 g) was isolated from each individual Danish Yorkshire Landrace pig (*n* = 3). Tissues were suspended in 10× w/v sucrose buffer and homogenised using a teflon glass homogeniser (20 strokes, on ice). The crude homogenate was centrifuged at a low speed (1000 *g*, 10 min, 4 °C) to generate the P1. The supernatant (S1) was set aside for further centrifugation. P1 was re-suspended in Tris buffer and washed twice by centrifugation at (1000 *g*, 10 min, 4 °C), with the resulting additional S1 supernatants added to the original supernatant (S1). The total S1 was centrifuged at a higher speed (17,000 *g*, 20 min, 4 °C) to generate P2. The supernatant resulting from this spin (S2) was set aside for further centrifugation. P2 pellet was re-suspended in Tris buffer and washed twice by centrifugation (32,000 *g*, 20 min, 4 °C), with the resulting additional S2 supernatants added to the original supernatant (S2). The total S2 was centrifuged (100,000 *g*, 90 min, 4 °C) to generate the P3. The supernatant from P3, S3, was collected as the cytosolic fraction of the striatal cells. P2 was resuspended in Extracellular buffer (see below). P3 was resuspended in endosomal buffer (see below). Samples of the cell fractions (250 μl of each) were assessed for protein content and the remainder aliquoted and stored at −80 °C for use in subsequent radioligand binding studies.

### 2.2 Radioligand Binding assays

#### 2.2.1 Saturation studies

Tissue samples (P2) generated from rat striatal membranes were diluted to 200 μg membrane protein/well in each of the three relevant physiological buffers, previously reported by Quelch et al. 2012 (1) Extracellular (Extra = 50 mM Tris HCl, 140 mM NaCl, 5 mM KCl, 1.5 mM MgCl_2_, 1.5 mM CaCl_2_, pH 7.4, 37 °C); (2) Intracellular (Intra = 50 mM Tris HCl, 10 mM NaCl, 140 mM KCl, 0.5 mM MgCl_2_, pH 7.0, 37 °C) and (3) Endosomal (Endo = 20 mM MES, 10 mM NaCl, 140 mM KCl, 0.5 mM MgCl_2_, 0.003 mM CaCl_2_, pH 6.0, 37 °C) (Quelch et al., 2012); concentrations are adapted from Brinley (1980), Murphy et al. (1984), Gerasimenko et al. (1998), Gekle et al. (1999), Grabe and Oster (2001), Alberts et al. (2002), Christensen et al. (2002), Faundez and Hartzell (2004), Somjen (2004) and incubated (37 °C) with a range of radioligand concentrations ([^3^H]raclopride 0.003–30 nM, [^3^H]PhNO 0.003–10 and [^3^H]spiperone 0.003–10 nM). The specific binding component was determined using haloperidol (1 μM). Assays were terminated via filtration through Whatman GF/B filters followed by 4 × 1 ml washes with ice-cold Wash buffer (50 mM Tris HCl, pH 7.4, 4 °C). Filters were transferred to scintillation vials and scintillation fluid (3 ml/vial; Packard Ultima Gold MV) added and bound radioactivity determined on a Packard Tricarb liquid scintillation counter. Whatman GF/B filters were pre-incubated in polyethylenimine (PEI; 0.05%, 60 min) prior to filtration.

#### 2.2.2 Sub-cellular fraction studies

Radioligand binding studies were performed from three independent fractionation procedures using pig striata. Fractions, P2, P3endo and S3, were diluted to 200 μg protein/well for addition to assay. These studies were performed using fixed concentrations of [^3^H]spiperone (5 nM), [^3^H]raclopride (20 nM) and [^3^H]PhNO (5 nM). The specific binding component was determined using haloperidol (1 μM). Following radioligand addition, assays were incubated at 37 °C (60 min). Assays were terminated via filtration and radioactivity counted as described above.

#### 2.2.3 Kinetic studies

Rat striatal P2 homogenates were thawed and diluted to the desired concentration (200 μg membrane protein/well) in both extracellular and endosomal buffers. For association experiments, [^3^H]spiperone (1 nM), [^3^H]raclopride (2 nM) or [^3^H]PhNO (1 nM) were added at 22 time points between 0 and 100 min to wells containing 400 μl protein and 50 μl assay buffer. For dissociation studies, protein and [^3^H]spiperone (1 nM), [^3^H]raclopride (2 nM) or [^3^H](+)PhNO (1 nM) were added to the assay plate and allowed to associate at 37 °C for 100 min. Following this, haloperidol (1 μM) was added to the plate at 22 time points between 101 and 200 min, post-association in order to begin the dissociation. All time points were terminated simultaneously via filtration and radioactivity counted as described above.

### 2.3 Protein assay

Protein concentrations were determined by the colorimetric method using bicinchoninic acid assay at 562 nm (Thermo Scientific Pierce BCA Protein Assay Kit) (Smith et al., 1985).

### 2.4 Data analysis

All data were analysed using GraphPad Prism 5.0. One- and Two-way ANOVA were performed using SigmaStat 3.0. Student's *t*-tests were performed using GraphPad Prism 5.0. B_*max*_ values in pmol/g tissue (nM) were generated according to Equation (1) (where it is assumed that 1 ml of homogenised wet weight tissue is equivalent to 1 g in weight). *In vitro* binding potentials (BP) were generated using Equation (2). Relative Specific Activity (RSA) values were generated using Equation (3) (Laduron, 1977). Kinetic parameters, k_on_ and K_*D*_ values were determined using Equations (4) and (5), respectively.

B_*max*_ in pmol/g tissue determination (where pmol/g is equivalent to nM);

(1) $${\text{B}}_{max}^{'}=\frac{{\text{B}}_{max}\times \text{Protein}\;\text{content}}{\text{Mass}\;\text{tissue}}$$

Where ${\text{B}}_{max}^{'}$ is the maximum number of binding sites expressed in pmol/g tissue equivalent to nM; B_*max*_ is the maximum number of binding sites expressed in fmol/mg of protein; “Mass tissue” in assay in g/ml; and “Protein content” in mg/ml and it is assumed that 1 g protein is the equivalent to 1 ml.

*In vitro* BP determination;

(2) $$iv\text{BP}=\frac{{\text{B}}_{max}^{'}}{K_{d}}$$

Where *iv*BP is the *in vitro* binding potential (unit less); ${\text{B}}_{max}^{'}$ is the maximum number of binding sites expressed in pmol/g tissue equivalent to nM and *K*_*d*_ is the equilibrium dissociation constant in nM.

Relative specific activity (RSA) determination;

(3) $$\text{RSA}=\frac{\%\;\text{Specific}\;\text{binding}\;\text{per}\;\text{fraction}}{\%\;\text{Protein}\;\text{per}\;\text{fraction}}$$

Where RSA is the relative specific activity in each fraction; “% Specific binding per fraction” is the percentage of total specific binding of the radioligand in the fraction and “% Protein per fraction” is the percentage of the total protein in the fraction.

k_*on*_ determination;

(4) $$k_{on}=\frac{\left(k_{obs}-k_{off}\right)}{\left[\text{radioligand}\right]}$$

Where *k*_*on*_ is the rate constant of the association of radioligand and receptor in M^−1^ min^−1^; *k*_*obs*_ is the observed rate constant for the association of radioligand and receptor in min^−1^; *k*_*off*_ is the rate constant for the dissociation of radioligand and receptor in min^−1^ and [radioligand] is the concentration of radioligand in M.

Kinetic K_*D*_ determination;

(5) $$K_{d}=\frac{k_{off}}{k_{on}}$$

Where *K*_*d*_ is the equilibrium dissociation constant in M; *k*_*off*_ is the rate constant for the dissociation of radioligand and receptor in min^−1^ and *k*_*on*_ is the rate constant of the association of radioligand and receptor in M^−1^ min^−1^.

## 3 Results

### 3.1 Cellular environments

#### 3.1.1 Saturation and kinetic studies – [^3^H]raclopride and [^3^H]PhNO

For all K_*D*_ and B_*max*_ determinations plateaus were reached within the concentration ranges tested. The data from all conditions and radioligands tested were best fit to a single site. In general, changing the physiological environments had no effect on the observed B_*max*_ for either [^3^H]raclopride or [^3^H]PhNO (Table 1). A significant reduction in K_*D*_ for both [^3^H]PhNO and [^3^H]raclopride was observed in the endosomal condition compared with the extracellular condition (Table 1). Additionally, [^3^H]raclopride and [^3^H]PhNO kinetic K_*D*_ values obtained in the extracellular conditions were comparable with those obtained from saturation studies (Table 2). k_*off*_ rates for [^3^H]raclopride, and accurate k_*ob*_ values for [^3^H]PhNO were unable to be determined in the endosomal condition, with full inhibition of binding and association plateau not being achieved after 6 h following assay initiation (Table 2).

#### 3.1.2 Saturation and kinetic studies – [^3^H]spiperone

A significant effect of cellular environment was observed for K_*D*_ values obtained with [^3^H]spiperone (Table 1), however the magnitude of this change in K_*D*_ was less than that compared with [^3^H]raclopride or [^3^H]PhNO. The kinetic K_*D*_ obtained for [^3^H]spiperone was slightly lower (non-significant) than that determined from saturation studies (Table 2). In addition, the kinetic K_*D*_ determined in the extracellular condition was comparable to that in the endosomal condition. An increase in association rate was observed in the extracellular compared to the endosomal environment for [^3^H]spiperone; this was not significant (Table 2).

#### 3.1.3 Saturation and kinetic studies – *in vitro* BPs

*In vivo* PET data is generally reported in terms of binding potential (BP ∝ B_*max*_/K_*d*_). *In vitro* BPs were generated using Equation (2) (Table 1). As expected from the saturation K_*D*_ values, a significant reduction in *in vitro* BP values were observed with [^3^H]raclopride, [^3^H]PhNO and [^3^H]spiperone in the endosomal environment compared to the extracellular (Table 1), therefore the following trend for the reduction in ‘Percentage of Extracellular *in vitro* BP’ in the endosomal condition was observed: [^3^H]PhNO > [^3^H]raclopride > [^3^H]spiperone (Table 1).

### 3.2 Sub-cellular fractionation

The greatest amount of striatal cell protein was observed in the cytosolic fraction (Table 3). Lower levels were observed in the plasma membrane and microsomal fractions (Table 3).

For each of the three radioligands investigated, the majority of “Percent Bound per Fraction” was observed in the P2 fraction (∼67–83%) with lower levels bound to the P3 fraction (∼14–25%) and much lower levels bound to the S3 fraction (∼3–8%; Table 3). No significant differences in plasma membrane, microsomal or cytosolic percentage bound per fraction were observed when comparing the cellular distribution across the three radioligands (Table 3).

**Fig. 1:**
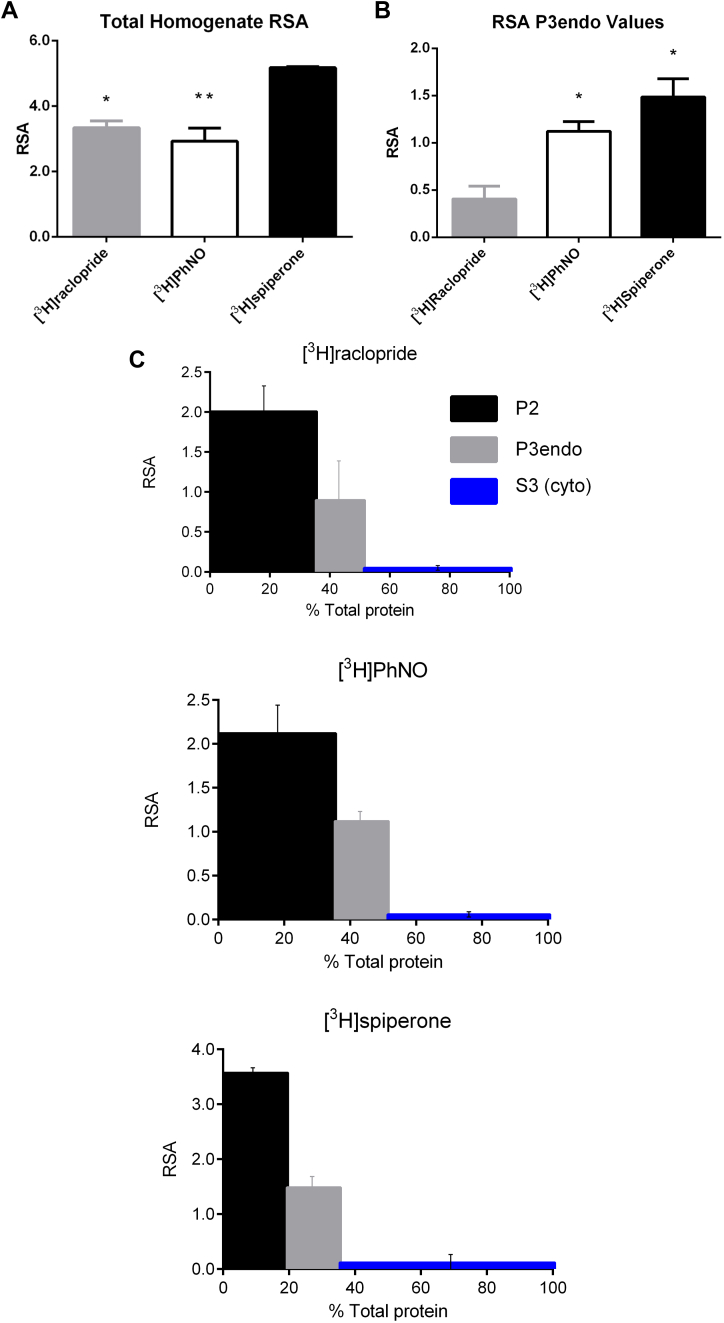
**A.** Comparison of [^3^H]raclopride, [^3^H]PhNO and [^3^H]spiperone “Total Homogenate RSA values”. Total homogenate binding observed following sub-cellular fractionation of pig striatal membranes; *n* = 3 for [^3^H]raclopride and [^3^H]PhNO; *n* = 2 for [^3^H]spiperone (mean ± s.e.mean). One-way ANOVA with Tukey post-test were performed using SigmaStat 3.0. ^∗^*p* < 0.05 and ^∗∗^*p* < 0.01 represent comparison of [^3^H]spiperone RSA to both [^3^H]raclopride and [^3^H]PhNO. **B.** Microsomal fraction RSA values with [^3^H]raclopride, [^3^H]PhNO and [^3^H]spiperone. [^3^H]Raclopride and [^3^H]PhNO *n* = 3; *n* = 2 for [^3^H]spiperone (mean ± s.e.mean). ^∗^*p* < 0.05 represent comparison of [^3^H]raclopride P3 RSA with [^3^H]spiperone and [^3^H]PhNO P3 RSA. **C.** Representation of homogenate binding as a function of cell protein. Relative specific activity (RSA) versus percent total protein per fraction histogram for [^3^H]raclopride, [^3^H](+)PhNO and [^3^H]spiperone from pig striatal sub-cellular fractionation; *n* = 3 for [^3^H]raclopride and [^3^H]PhNO; *n* = 2 for [^3^H]spiperone (mean ± s.e.mean).

When both “Percentage Bound per Fraction” and “Percentage Total Protein per Fraction” are both taken into account i.e. using Equation (3), relative specific activity (RSA) values are generated. The total striatal homogenate RSA values for [^3^H]spiperone were found to be significantly greater than those obtained for both [^3^H]raclopride and [^3^H]PhNO (Fig. 1A, Table 3). Similar to the distribution of “Percentage total homogenate Bound per Fraction”, RSA distribution was highest in the P2-plasma membrane fraction for all ligands (Table 3).

No significant differences were observed between [^3^H]raclopride, [^3^H]PhNO and [^3^H]spiperone P2 and S3 RSA values. However a significantly greater RSA value was present in P3 fractions with [^3^H]PhNO and [^3^H]spiperone when compared with [^3^H]raclopride (Fig. 1B, Table 3). A representation of striatal tissue fraction binding as a function of fraction protein content can be seen in Fig. 1C.

## 4 Discussion

We have sought to determine the effects of three physiological environments reflective of the cellular compartments experienced by a receptor following agonist-induced internalisation, on the binding parameters of the D_2/3_ radioligands [^3^H]raclopride, [^3^H]PhNO and [^3^H]spiperone. The relative contribution of each of these three cellular compartments (extracellular, intracellular and endosomal) to the overall basal PET signal given by the PET ligands raclopride, PhNO and spiperone have also been estimated. Using *in vitro* radioligand binding, a reduction in affinity was observed for [^3^H]raclopride and [^3^H]PhNO in the endosomal compared with the extracellular environment; two radioligands with known sensitivity to endogenous release *in vivo*. Alterations in receptor kinetics in the different cell conditions may underpin these changes in affinity. Affinity shifts with changing cellular environment lead to a reduction in *in vitro* BP generated for these radioligands, the magnitude of which was related to the change in BP following endogenous dopamine release observed previously *in vivo* with these ligands. Notably, compared with [^3^H]raclopride and [^3^H]PhNO, a smaller change in affinity was observed in the endosomal condition compared with the extracellular condition for [^3^H]spiperone, a radioligand thought not to be sensitive to endogenous dopamine release *in vivo*. These data suggest that changes in receptor affinity following dopamine release and subsequent agonist-induced internalisation may contribute to reductions in BP observed *in vivo*, since BP is proportional to B_*max*_/K_*D*_. Using sub-cellular fractionation we estimated that in the absence of a pharmacological challenge, the majority (∼67–∼83%) of the total signal associated with [^3^H]raclopride, [^3^H]PhNO and [^3^H]spiperone would result from membrane bound D_2/3_ receptors. In agreement with our saturation data we also demonstrate that ∼25% of total striatal binding for [^3^H[spiperone originates from sub-cellular, microsomal receptors, whereas for [^3^H]raclopride and [^3^H]PhNO, this fraction is lower, representing ∼14% and 17%, respectively. The cytosolic fraction contributes negligibly to the overall PET signal.

### 4.1 Effect of physiological environments on the binding properties of [^3^H]raclopride, [^3^H]PhNO and [^3^H]spiperone

Receptor availabilities (B_*max*_) and affinity (K_*D*_) values for all three radioligands were generated in the extracellular environment and found to be comparable to those previously reported in the literature (Seeman et al., 2007; Cumming, 2011). In order to recreate the cellular environments present at each of the sub-cellular and cell surface compartments as accurately as possible, a variety of literature sources were consulted that have examined the ionic compositions of various cell compartments and organelles (Brinley, 1980; Murphy et al., 1984; Gerasimenko et al., 1998; Gekle et al., 1999; Grabe and Oster, 2001; Alberts et al., 2002; Christensen et al., 2002; Faundez and Hartzell, 2004; Somjen, 2004). These cellular buffers have previously been used to investigate the effects of serotonin transporter endocytosis and sub-cellular localisation on the binding of [^3^H]DASB (Quelch et al., 2012). To further validate the translation from the effects of the cellular buffers on surface receptors in an endosomal condition to endosomal receptors (found in the P3 fraction) we have performed P3 binding in both extracellular and endosomal conditions. With [^3^H]raclopride, for example, P3 binding is 1.98 fold higher in the extracellular condition (RSA = 1.77) compared with when conducted in the endosomal condition (RSA = 0.89). Interpolating from the saturation binding curves conducted in P2 homogenates in different cellular buffers at the radioligand concentration used in the fractionation binding studies (20 nM for [^3^H]raclopride), the specific binding in the extracellular condition is 1.95 fold greater in the extracellular condition (331.4 fmol/mg) compared with the endosomal condition (170.5 fmol/mgn). We consider these binding shifts very similar and therefore assume these are representative of the affinity shift experienced by endosomally trafficking receptors.

The effects of changing sodium levels on receptor affinity for benzamide ligands, e.g. raclopride, and butyrophenone ligands, e.g. spiperone have previously been investigated (Stefanini et al., 1980; Freedman et al., 1982; Neve, 1991; Neve et al., 1991; D'Souza and Strange, 1995). However, the effects of these changing ionic conditions have been less extensively investigated for the D_3_-preferring agonist ligand, [^3^H]PhNO. Additionally, the effect of changing the ionic composition of not just sodium but also pH, calcium, potassium, magnesium and chloride ions to concentrations representative of those found in the extracellular, intracellular and endosomal compartments on the specific binding of the three D_2/3_ ligands have also not been reported previously.

In agreement with the saturation data presented here, Neve (1991) demonstrated a decrease in benzamide ([^125^I]epidepride), but not butyrophenone ligand ([^3^H]spiperone), affinity following a reduction in assay pH and sodium concentration. Two amino-acid sites within the D_2_ are thought to confer benzamide pH and sodium sensitivity with a pK_*a*_ of 6–7, whereas one site with a pK_*a*_ of 5.5–6.1 is thought to confer this sensitivity for butyrophenone ligands (D'Souza and Strange, 1995). Within our physiological buffers, both amino-acids required for benzamide binding could be ionised at pH6.0 within the endosomal condition leading to the reduction in [^3^H]raclopride binding. This pH of 6.0 may not be acidic enough to cause the ionisation of the single site required for [^3^H]spiperone binding, which would account for [^3^H]spiperone appearing less sensitive to the different conditions tested in this study. [^3^H]PhNO was more sensitive to changes in ionic environment than the D_2/3_ receptor antagonist radioligands, suggesting that the endosomal environment may be less favourable for D_2_^high^ receptor than for D_2_^low^ receptor binding. Inclusion of magnesium and calcium ions in assay buffers has been shown to increase affinity of agonist ligands such as NPA and apomorphine (Sibley and Creese, 1983; Abolfathi and Di Paolo, 1991). However, no significant reduction in affinity was observed from extracellular to intracellular (where magnesium and calcium were both depleted compared to the extracellular) but when, pH was further dropped from 7.0 to 6.0 (and magnesium and calcium were still depleted), [^3^H]PhNO affinity was significantly reduced. Therefore the dramatic loss of affinity observed by [^3^H]PhNO appears to be mainly pH driven. Together, these data further support the idea that agonist PET ligands may be preferential for use when trying to image endogenous release *in vivo* since they would be expected to exhibit a greater loss of receptor affinity and hence a greater decrease in *in vivo* BP following endogenous transmitter release and translocation to the endosomal compartment.

Guo et al. (2010) have demonstrated a similar affinity shift following quinpirole induced D_2_ receptor internalisation of raclopride, PhNO and spiperone in D_2short_ expressing cell systems. Building upon these findings, we suggest that following internalisation of D_2/3_ receptors to endosomal vesicles, the decrease in affinity observed may be driven by alterations in receptor binding kinetics. For both [^3^H]raclopride and [^3^H]PhNO, two ligands known to be sensitive to endogenous release of dopamine *in vivo*, we show incomplete equilibrium kinetics in the endosomal compartment compared with that observed at the plasma membrane. Furthermore, no significant changes in the kinetic parameters measured were observed between extracellular and endosomal cellular environments with [^3^H]spiperone. These data support, both homogenate binding studies and *in vivo* dosing data, which suggest [^3^H]spiperone binding is less sensitive to changes in sodium and hydrogen ion concentration and intracellular trapping of [^3^H]spiperone following endocytosis of D_2/3_ receptors (Chugani et al., 1988). Importantly, we report the change in *in vitro* BP from the extracellular to the endosomal condition is consistent with the known sensitivity to endogenous dopamine release previously observed *in vivo* i.e. [^11^C/^3^H]PhNO > [^11^C/^3^H]raclopride > [^11^C/^3^H]spiperone (Hartvig et al., 1997; Shotbolt et al., 2011). These findings suggest that in addition to different pharmacological properties of radioligands (i.e. antagonist versus agonist radioligands) contributing to sensitivity to endogenous neurotransmitter release, a greater effect of receptor internalisation on radioligand binding parameters may also drive an increased vulnerability to signal changes observed *in vivo* with [^11^C/^3^H]PhNO compared with [^11^C/^3^H]raclopride or [^11^C/^3^H]spiperone. Investigating the sensitivity of other D_2/3_ radioligand's (such as NPA, fallypride and FLB 457), to the different environments used in our assays therefore now warrants further investigation.

### 4.2 Cellular composition of striatal binding for [^3^H]raclopride, [^3^H]PhNO and [^3^H]spiperone

The cellular and sub-cellular distribution of dopamine receptors have previously been described (Chugani et al., 1988; Yung et al., 1995; Bloch et al., 2003; Sun et al., 2003; Voulalas et al., 2011). We present relative specific activity values (RSA), which represent binding as a function of fraction protein content (Laduron, 1977). D_2_ expression has been observed previously in cytosolic fractions using semi-quantitative Western blot analysis (Voulalas et al., 2011) and electron microscopy (Paspalas et al., 2006). D_3_ receptor cytoplasmic and intracellular vesicle localisation has also been noted (Diaz et al., 2000). Together with the binding data from the cytosolic compartment (S3), this suggests that both D_2_ and D_3_ receptors may be present in the cytoplasm but may not be in the correct tertiary conformation necessary for radioligand binding, and hence contributes very little to total the tissue binding as a consequence. Despite the highest levels of binding emanating from the plasma membrane fractions for all three radioligands, the ‘Percent Bound Per Fraction’ obtained for P2 and P3 for [^3^H]raclopride, [^3^H](+)PhNO and [^3^H]spiperone suggests that a significant proportion of the signals observed *in vivo* could be due to subcellular, microsomal binding. The RSA values for both [^3^H]spiperone and [^3^H]PhNO in the microsomal fraction were greater than that observed for [^3^H]raclopride. This may represent an increased abundance of D_3_ receptor in the sub-cellular compartments which would lead to increases in [^3^H]PhNO binding in the microsomal compartments compared with [^3^H]raclopride. Increased [^3^H]spiperone binding in microsomal fractions maybe due to sequestered levels of 5HT_2A_ receptors. [^3^H]Spiperone has been shown to have significant affinity at 5HT_2A_ receptors (∼0.5 nM in transfected HEK293 cells) (Pritchett et al., 1988). Furthermore, the majority 5HT_2A_ receptors located on dendrites of rat ventral tegmental area dopaminergic cells have been shown to be sub-cellularly located (Doherty and Pickel, 2000).

In this manuscript we describe the cellular composition of total striatal tissue binding with [^3^H]raclopride, [^3^H]PhNO and [^3^H]spiperone. Laruelle (2012) recently proposed an updated D_2_ receptor model with reference to D_2/3_ PET imaging, whereby ∼60% of striatal D_2/3_ are configured in a high affinity state and ∼35% are configured in a low affinity state (based on studies comparing agonist versus antagonist D_2/3_ PET ligands) (Laruelle, 2012). We demonstrate here a population of receptors, similar in ‘Percent Bound Per Fraction’ across all three radioligands that represents D_2/3_ at the plasma membrane (∼67–∼83%). This population is comparable in magnitude to that described by Laruelle (2012) when combining both D_2/3_^high/low^ but excluding a population of extrasynaptic or internalised D_2/3_ receptors (∼75% (Laruelle, 2012)). The studies presented here do not distinguish between synaptic and extrasynaptic D_2/3_, however we propose that despite the similarities in proportions of membrane bound receptors (comprising the majority of total striatal binding for all three radioligands tested) the differences in their behaviours following endogenous dopamine release *in vivo* will possibly be dependent on their ability to bind D_2/3_ following internalisation. This is reflected using both saturation studies conducted in different cellular environments and sub-cellular fractionation studies where for [^3^H]raclopride and [^3^H]PhNO, the microsomal fraction contribution to total striatal binding (∼14% and 17%, respectively) is considerably smaller compared with that of [^3^H]spiperone's (∼25%). Sub-cellular fractionation following pharmacological challenge remains to be conducted using the methodology implemented in this manuscript. However, we demonstrate using the percent bound per fraction for each radioligand and the affinity values for each ligand in each physiological buffer, the expected magnitude of signal change should a constant amount of membrane D_2/3_ endocytose to the microsomal compartment (20% internalisation is used an example, see Table 4). These data suggest that following endocytosis, the total striatal binding capacity for [^3^H]raclopride, [^3^H]PhNO and [^3^H]spiperone would be reduced in the same rank order as that seen *in vivo* with these ligands following administration of a dopamine releasing agent i.e. [^3^H]PhNO (17.2%) > [^3^H]raclopride (14.5%) > [^3^H]spiperone (11.5%). These data remain to be corroborated in a pharmacological challenge model using the methodology implemented here. We believe this would be of benefit to furthering our understanding of the cellular mechanisms underpinning signal changes observed *in vivo* not only with D_2/3_ receptors but, also other receptor radioligands sensitive to change following endogenous neurotransmitter release.

In summary, using *in vitro* radioligand binding studies, we have demonstrated that a reduction in affinity was observed for all ligands tested in the endosomal compared with the extracellular environment. The magnitude of this affinity shift with changing cellular environment was related to the change in BP reported previously *in vivo* with these ligands i.e. [^3^H]raclopride and [^3^H]PhNO displayed a much greater reduction in binding in the endosomal condition compared with [^3^H]spiperone, a radioligand thought not to be sensitive to endogenous dopamine release *in vivo*. Therefore, we suggest that assessment of these affinity shifts predicts ligand sensitivity to endogenous dopamine release. Using sub-cellular fractionation assays we have estimated that in the absence of a pharmacological challenge, the majority (∼67–∼83%) of the total signal associated with [^3^H]raclopride, [^3^H]PhNO and [^3^H]spiperone would result from membrane bound D_2/3_ receptors; with a significant (∼14–∼25%) contribution being derived from microsomal D_2/3_ receptors and the cytosolic fraction contributing negligibly to the overall PET signal.

This novel approach for estimating the cellular composition of a PET signal under basal conditions now warrants further use for other receptor PET ligands. Determination of susceptibility to affinity shifts following receptor internalisation may be a crucial radioligand characteristic for determining sensitivity to neurotransmitter release *in vivo*. As such, the data presented here may help translate *in vivo* competition imaging techniques to neurotransmitter systems outside the D_2/3_. Translational approaches such as this, allow for an enhanced understanding of the pharmacology underlying the *in vivo* PET signals for multiple receptor systems, from both endogenous transmitter release and exogenous challenge studies.

## Funding and disclosure

The studies presented here were financially supported by a BBSRC and GSK CASE studentship award (BB/H53072X/1).

## Figures and Tables

**Figure:**
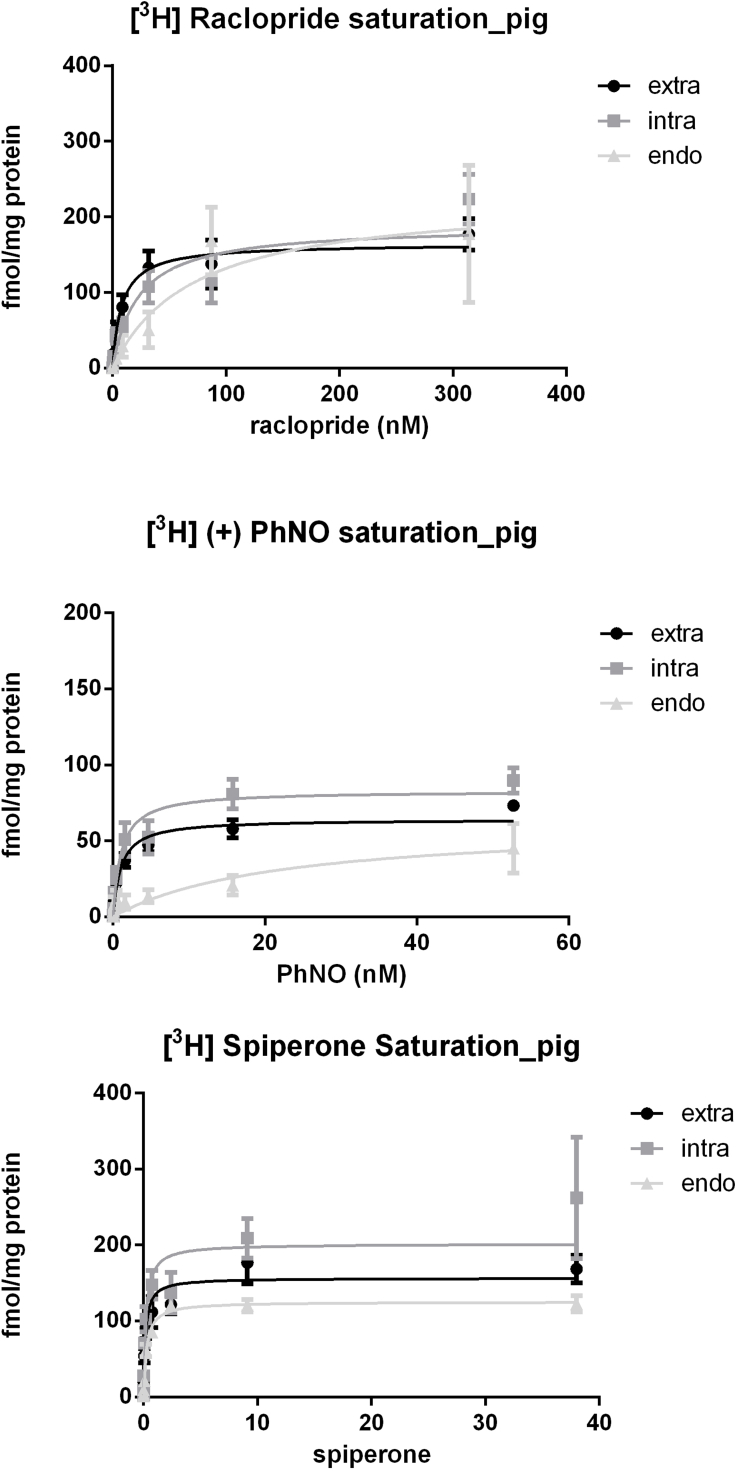


**Table 1:** K_*D*_, B_*max*_ and *in vitro* BP values for each radioligand in the three physiological environments.

Radioligand and buffer		K_*D*_ (nM)	B_*max*_ (fmol/mg protein)	B_*max*_ (pmol/g tissue)	*In vitro* BP	% of extra *in vitro* BP_p_
[^3^H]Raclopride						
	Extra	2.0 ± 0.2	368 ± 80	13.8 ± 0.8	6.9 ± 0.1	100
	Intra	4.3 ± 0.8	381 ± 86	12.9 ± 0.7	3.2 ± 0.3	47
	Endo	9.5 ± 3.6^∗∗^	292 ± 68	9.1 ± 0.5	1.2 ± 0.3^∗∗^	18
[^3^H]PhNO						
	Extra	0.6 ± 0.1	130 ± 21	7.2 ± 0.2	14.5 ± 4.1	100
	Intra	0.3 ± 0.1	169 ± 32	8.2 ± 0.5	25.4 ± 5.7	174
	Endo	10.3 ± 2.7^∗∗^	103 ± 41	6.1 ± 1.6	0.6 ± 0.1^∗∗^	4
[^3^H]Spiperone						
	Extra	0.1 ± 0.01	265 ± 39	15.2 ± 0.5	179.5 ± 28.1	100
	Intra	0.1 ± 0.1	349 ± 35	18.6 ± 1.6	168.1 ± 10.9	93
	Endo	0.4 ± 0.2^∗^	278 ± 51	18.3 ± 1.9	68.43 ± 14.2^∗^	38

K_*D*_ (nM) and B_*max*_ (fmol/mg protein and pmol/g tissue) and *in vitro* BP values for [^3^H]raclopride, [^3^H](+)PhNO and [^3^H]spiperone in rat striatum (*n* = 4, mean ± s.e.mean) in the three ionic environments (Extra, Intra and Endo). One-way ANOVA with Tukey post-test were performed using SigmaStat 3.0. ^∗^*p <* 0.05 and ^∗∗^*p <* 0.01 represent comparison of intracellular or endosomal to the extracellular condition. ANOVA results. [^3^H]Raclopride, K_*D*_: *F*(3,20) = 12.47, *p* < 0.07; B_*max*_: *F*(2,20) = 3.40, *p* = 0.053. [^3^H]PhNO, K_*D*_: *F*(2,21) = 5.95, *p* < 0.009; B_*max*_: *F*(2,20) = 1.30, *p* = 0.03. [^3^H]Spiperone, K_*D*_: *F*(2,22) = 3.28, *p* < 0.057; B_*max*_: *F*(2,22) = 2.76, *p* < 0.085.

**Table 2:** Kinetic values for radioligands in extracellular and endosomal conditions.

Radioligand and buffer		k_*off*_ (min^−1^)	k_*ob*_ (min^−1^)	k_*on*_ (M^−1^ min^−1^)	Kinetic K_*D*_ (nM)	Saturation K_D_ (nM)	Fold difference^$^
[^3^H]Raclopride							
	Extra	0.4 ± 0.02	1.1 ± 0.2	0.5 ± 0.3	1.1 ± 0.5	2.0 ± 0.2	1.8 ± 1.1
	Endo	ND	0.03 ± 0.01	ND	−/−	9.5 ± 3.6	−/−
[^3^H]PhNO							
	Extra	0.1 ± 0.02	0.6 ± 1.6	0.4 ± 0.3	0.7 ± 0.1	0.6 ± 0.1	0.9 ± 0.1
	Endo	0.1 ± 0.1	ND	ND	−/−	10.3 ± 2.7	−/−
[^3^H]Spiperone							
	Extra	0.1 ± 0.01	0.4 ± 0.2	0.4 ± 0.2	0.6 ± 0.5	0.1 ± 0.01	0.2 ± 0.02
	Endo	0.1 ± 0.03	0.2 ± 0.02	0.1 ± 0.04	1.0 ± 0.5	0.4 ± 0.2	0.4 ± 0.4

k_*off*_ (min^−1^), k_*ob*_ (min^−1^), k_*on*_ (M^−1^ min^−1^), Kinetic K_*D*_ (nM) and Saturation K_*D*_ (nM)/Kinetic K_*D*_ (nM) fold difference for [^3^H]raclopride (*n* = 3 extra, *n* = 5 endo), [^3^H](+)PhNO (*n* = 4 extra, *n* = 2 endo) and [^3^H]spiperone (*n* = 3 extra, *n* = 3 endo; all data are mean ± s.e.mean). ^$^Represent Saturation K_*D*_/Kinetic K_*D*_. All data analysed with GraphPad Prism 5.0 using single site exponential association and dissociation fits.

**Table 3:** Sub-cellular fractionation data from pig striatal membranes.

Radioligand and fraction		% Total protein per fraction	% Bound per fraction	Homogenate RSA
[^3^H]Raclopride				
	P2	28.9 ± 4.5	82.5 ± 7.4	2.0 ± 0.3
	P3	15.6 ± 1.3	14.3 ± 7.5	0.9 ± 0.5
	S3	55.5 ± 4.9	3.2 ± 1.8	0.1 ± 0.03
				*Total* = 3.0^*^
[^3^H]PhNO				
	P2	28.9 ± 4.5	79.6 ± 2.5	2.1 ± 0.3
	P3	15.6 ± 1.3	17.3 ± 2.6	1.1 ± 0.1
	S3	55.5 ± 4.9	3.2 ± 1.7	0.1 ± 0.03
				*Total* = 3.3^**^
[^3^H]Spiperone				
	P2	18.1 ± 1.3	67.2 ± 2.9	3.6 ± 0.1
	P3	16.1 ± 2.7	24.5 ± 7.2	1.5 ± 0.2
	S3	65.1 ± 4.0	8.3 ± 10.1	0.1 ± 0.2
				*Total* = *5.2*

Percentage of total amount protein per fraction, Percentage of total amount of [^3^H]raclopride [^3^H](+)PhNO and [^3^H]spiperone bound to each fraction (P2, P3 and S3), relative specific activity (RSA) and Percentage of total binding signal (where 100% = P2 + P3 + S3) for [^3^H]raclopride, [^3^H](+)PhNO and [^3^H]spiperone from pig striatal membrane fractionation studies (*n* = 3, mean ± s.e.mean). ^∗^*p* < 0.05 and ^∗∗^*p* < 0.01 represent comparison of total homogenate RSA. Total homogenate RSA. [^3^H]Raclopride and [^3^H[PhNO binding were performed on the same striatal homogenate fractionation preparations, [^3^H]spiperone binding was performed on a separate set of striatal preparations. ANOVA results *F*(2,5) = 13.49, *p* = 0.0097. P3 RSA ANOVA results: *F*(2,4) = 13.55, *p* = 0.017.

**Table 4:** Prediction of alteration in total binding following D_2/3_ endocytosis.

Radioligand	K_*D*_ (nM)	% Bound per fraction	Fraction binding capacity (% binding per fraction/K_*D*_)	20% of surface receptors endocytose – % binding/fraction^€^	Fraction binding capacity post-endocytosis (% binding per fraction/K_*D*_)
[^3^H]Raclopride					
Cell Surface	2.0 (extra)	82.5 (P2)	41.3	66.0	33.0
Microsomal	9.5 (endo)	14.3 (P3)	1.5	32.8	3.5
Cytosol	4.3 (intra)	3.2 (S3)	0.7	3.2	0.7
			*Total: 43.5*		*Total: 37.2****(14.5% reduction)***
[^3^H]PhNO					
Cell Surface	0.6 (extra)	79.6 (P2)	132.7	63.7	106.2
Microsomal	10.3 (endo)	17.3 (P3)	1.68	33.2	3.3
Cytosol	0.3 (intra)	3.2 (S3)	10.7	10.7	10.7
			*Total: 145.0*		*Total: 120****(17.2% reduction)***
[^3^H]Spiperone					
Cell Surface	0.1 (extra)	67.2 (P2)	672.1	53.8	538
Microsomal	0.4 (endo)	24.5 (P3)	81.7	37.0	126.3
Cytosol	0.1 (intra)	8.3 (S3)	21.5	8.3	21.5
			*Total: 775.2*		*Total: 685****(11.5% reduction)***

Prediction of alteration in total binding following D_2/3_ endocytosis. ^€^Assumes that 20% of surface, membrane bound receptors endocytose to the microsomal compartment, and that no lysosomal trafficking and degradation of existing receptors occurs.

## References

[bib1] Abi-Dargham A. (2004). Do we still believe in the dopamine hypothesis? New data bring new evidence. Int. J. Neuropsychopharmacol..

[bib2] Abolfathi Z., Di Paolo T. (1991). Modulation of dopamine receptor agonist binding sites by cations and estradiol in intact pituitary and 7315a tumors. Biochem. Pharmacol..

[bib3] Alberts B., Johnson A., Lewis J., Raff M., Roberts K., Walker P. (2002). Molecular Biology if the Cell.

[bib4] Bloch B., Bernard V., Dumartin B. (2003). “In vivo” intraneuronal trafficking of G protein coupled receptors in the striatum: regulation by dopaminergic and cholinergic environment. Biol. Cell/Under Auspices Eur. Cell Biol. Organ..

[bib5] Brinley F.J. (1980). Regulation of intracellular calcium in squid axons. Fed. Proc..

[bib6] Cardenas L., Houle S., Kapur S., Busto U.E. (2004). Oral D-amphetamine causes prolonged displacement of [11C]raclopride as measured by PET. Synapse.

[bib7] Cho E.Y., Cho D.I., Park J.H., Kurose H., Caron M.G., Kim K.M. (2007). Roles of protein kinase C and actin-binding protein 280 in the regulation of intracellular trafficking of dopamine D3 receptor. Mol. Endocrinol..

[bib8] Christensen K.A., Myers J.T., Swanson J.A. (2002). pH-dependent regulation of lysosomal calcium in macrophages. J. Cell Sci..

[bib9] Chugani D.C., Ackermann R.F., Phelps M.E. (1988). In vivo [3H]spiperone binding: evidence for accumulation in corpus striatum by agonist-mediated receptor internalization. J. Cereb. Blood Flow Metab. Off. J. Int. Soc. Cereb. Blood Flow Metab..

[bib10] Cumming P. (2011). Absolute abundances and affinity states of dopamine receptors in mammalian brain: a review. Synapse.

[bib11] D'Souza U.M., Strange P.G. (1995). pH dependence of ligand binding to D2 dopamine receptors. Biochemistry.

[bib12] Diaz J., Pilon C., Le Foll B., Gros C., Triller A., Schwartz J.C., Sokoloff P. (2000). Dopamine D3 receptors expressed by all mesencephalic dopamine neurons. J. Neurosci. Off. J. Soc. Neurosci..

[bib13] Doherty M.D., Pickel V.M. (2000). Ultrastructural localization of the serotonin 2A receptor in dopaminergic neurons in the ventral tegmental area. Brain Res..

[bib14] Dumartin B., Caille I., Gonon F., Bloch B. (1998). Internalization of D1 dopamine receptor in striatal neurons in vivo as evidence of activation by dopamine agonists. J. Neurosci. Off. J. Soc. Neurosci..

[bib15] Faundez V., Hartzell H.C. (2004). Intracellular chloride channels: determinants of function in the endosomal pathway. Sci. STKE Signal Transduct. Knowl. Environ..

[bib16] Freedman S.B., Poat J.A., Woodruff G.N. (1982). Influence of sodium and sulphydryl groups on [3H]sulpiride binding sites in rat striatal membranes. J. Neurochem..

[bib17] Gekle M., Drumm K., Mildenberger S., Freudinger R., Gassner B., Silbernagl S. (1999). Inhibition of Na+-H+ exchange impairs receptor-mediated albumin endocytosis in renal proximal tubule-derived epithelial cells from opossum. J. Physiol..

[bib18] Gerasimenko J.V., Tepikin A.V., Petersen O.H., Gerasimenko O.V. (1998). Calcium uptake via endocytosis with rapid release from acidifying endosomes. Curr. Biol. CB.

[bib19] Grabe M., Oster G. (2001). Regulation of organelle acidity. J. Gen. Physiol..

[bib20] Guo N., Guo W., Kralikova M., Jiang M., Schieren I., Narendran R., Slifstein M., Abi-Dargham A., Laruelle M., Javitch J.A., Rayport S. (2010). Impact of D2 receptor internalization on binding affinity of neuroimaging radiotracers. Neuropsychopharmacol. Off. Publ. Am. Coll. Neuropsychopharmacol..

[bib21] Hamblin M.W., Creese I. (1982). 3H-dopamine binding to rat striatal D-2 and D-3 sites: enhancement by magnesium and inhibition by guanine nucleotides and sodium. Life Sci..

[bib22] Hartvig P., Torstenson R., Tedroff J., Watanabe Y., Fasth K.J., Bjurling P., Langstrom B. (1997). Amphetamine effects on dopamine release and synthesis rate studied in the Rhesus monkey brain by positron emission tomography. J. Neural Transm..

[bib23] Houston G.C., Hume S.P., Hirani E., Goggi J.L., Grasby P.M. (2004). Temporal characterisation of amphetamine-induced dopamine release assessed with [11C]raclopride in anaesthetised rodents. Synapse.

[bib24] Kim K.M., Valenzano K.J., Robinson S.R., Yao W.D., Barak L.S., Caron M.G. (2001). Differential regulation of the dopamine D2 and D3 receptors by G protein-coupled receptor kinases and beta-arrestins. J. Biol. Chem..

[bib25] Kong M.M., Verma V., O'Dowd B.F., George S.R. (2011). The role of palmitoylation in directing dopamine D1 receptor internalization through selective endocytic routes. Biochem. Biophys. Res. Commun..

[bib26] Laduron P. (1977). Tissue fractionation in neurobiochemistry: an analytical tool or a source of artifacts. Int. Rev. Neurobiol..

[bib27] Laruelle M. (1998). Imaging dopamine transmission in schizophrenia. A review and meta-analysis. Q. J. Nucl. Med..

[bib28] Laruelle M. (2000). Imaging synaptic neurotransmission with in vivo binding competition techniques: a critical review. J. Cereb. Blood Flow Metab. Off. J. Int. Soc. Cereb. Blood Flow Metab..

[bib29] Laruelle M., Grunder G. (2012). Measuring dopamine synaptic transmission with molecular imaging and pharmacological challenges: the state of the art.

[bib30] Laruelle M., Iyer R.N., al-Tikriti M.S., Zea-Ponce Y., Malison R., Zoghbi S.S., Baldwin R.M., Kung H.F., Charney D.S., Hoffer P.B., Innis R.B., Bradberry C.W. (1997). Microdialysis and SPECT measurements of amphetamine-induced dopamine release in nonhuman primates. Synapse.

[bib31] Macey T.A., Gurevich V.V., Neve K.A. (2004). Preferential interaction between the dopamine D2 receptor and arrestin2 in neostriatal neurons. Mol. Pharmacol..

[bib32] Malmberg A., Mohell N. (1995). Characterization of [3H]quinpirole binding to human dopamine D2A and D3 receptors: effects of ions and guanine nucleotides. J. Pharmacol. Exp. Ther..

[bib33] Martin-Negrier M.L., Charron G., Bloch B. (2006). Receptor recycling mediates plasma membrane recovery of dopamine D1 receptors in dendrites and axons after agonist-induced endocytosis in primary cultures of striatal neurons. Synapse.

[bib34] Martinez D., Narendran R. (2010). Imaging neurotransmitter release by drugs of abuse. Curr. Topics Behav. Neurosci..

[bib35] Murphy J.F., Powers S., Cantor C.R. (1984). Endosome pH measured in single cells by dual fluorescence flow cytometry: rapid acidification of insulin to pH 6.0. J. Cell Biol..

[bib36] Narendran R., Slifstein M., Hwang D.R., Hwang Y., Scher E., Reeder S., Martinez D., Laruelle M. (2007). Amphetamine-induced dopamine release: duration of action as assessed with the D2/3 receptor agonist radiotracer (-)-N-[(11)C]propyl-norapomorphine ([11C]NPA) in an anesthetized nonhuman primate. Synapse.

[bib37] Neve K.A. (1991). Regulation of dopamine D2 receptors by sodium and pH. Mol. Pharmacol..

[bib38] Neve K.A., Cox B.A., Henningsen R.A., Spanoyannis A., Neve R.L. (1991). Pivotal role for aspartate-80 in the regulation of dopamine D2 receptor affinity for drugs and inhibition of adenylyl cyclase. Mol. Pharmacol..

[bib39] Paspalas C.D., Rakic P., Goldman-Rakic P.S. (2006). Internalization of D2 dopamine receptors is clathrin-dependent and select to dendro-axonic appositions in primate prefrontal cortex. Eur. J. Neurosci..

[bib40] Pritchett D.B., Bach A.W., Wozny M., Taleb O., Dal Toso R., Shih J.C., Seeburg P.H. (1988). Structure and functional expression of cloned rat serotonin 5HT-2 receptor. EMBO Journal.

[bib41] Quelch D.R., Parker C.A., Nutt D.J., Tyacke R.J., Erritzoe D. (2012). Influence of different cellular environments on [3H]DASB radioligand binding. Synapse.

[bib42] Seeman P., McCormick P.N., Kapur S. (2007). Increased dopamine D2^high^ receptors in amphetamine-sensitized rats, measured by the agonist [^3^Hl(+)PhNO. Synapse.

[bib43] Shotbolt P., Tziortzi A.C., Searle G.E., Colasanti A., van der Aart J., Abanades S., Plisson C., Miller S.R., Huiban M., Beaver J.D., Gunn R.N., Laruelle M., Rabiner E.A. (2011). Within-subject comparison of [(11)C]-(+)-PHNO and [(11)C]raclopride sensitivity to acute amphetamine challenge in healthy humans. J. Cereb. Blood Flow Metab. Off. J. Int. Soc. Cereb. Blood Flow Metab..

[bib44] Sibley D.R., Creese I. (1983). Regulation of ligand binding to pituitary D-2 dopaminergic receptors. Effects of divalent cations and functional group modification. J. Biol. Chem..

[bib45] Skinbjerg M., Liow J.S., Seneca N., Hong J., Lu S., Thorsell A., Heilig M., Pike V.W., Halldin C., Sibley D.R., Innis R.B. (2010). D2 dopamine receptor internalization prolongs the decrease of radioligand binding after amphetamine: a PET study in a receptor internalization-deficient mouse model. NeuroImage.

[bib46] Smith P.K., Krohn R.I., Hermanson G.T., Mallia A.K., Gartner F.H., Provenzano M.D., Fujimoto E.K., Goeke N.M., Olson B.J., Klenk D.C. (1985). Measurement of protein using bicinchoninic acid. Anal. Biochem..

[bib47] Sokoloff P., Andrieux M., Besancon R., Pilon C., Martres M.P., Giros B., Schwartz J.C. (1992). Pharmacology of human dopamine D3 receptor expressed in a mammalian cell line: comparison with D2 receptor. Eur. J. Pharmacol..

[bib48] Somjen G.G. (2004). Ions in the Brain, Normal Function, Seizures and Stroke.

[bib49] Stefanini E., Marchisio A.M., Devoto P., Vernaleone F., Collu R., Spano P.F. (1980). Sodium-dependent interaction of benzamides with dopamine receptors. Brain Res..

[bib50] Sun W., Ginovart N., Ko F., Seeman P., Kapur S. (2003). In vivo evidence for dopamine-mediated internalization of D2-receptors after amphetamine: differential findings with [3H]raclopride versus [3H]spiperone. Mol. Pharmacol..

[bib51] Tsao P., Cao T., von Zastrow M. (2001). Role of endocytosis in mediating downregulation of G-protein-coupled receptors. Trends Pharmacol. Sci..

[bib52] Volkow N.D., Fowler J.S., Wang G.J., Swanson J.M., Telang F. (2007). Dopamine in drug abuse and addiction: results of imaging studies and treatment implications. Arch. Neurol..

[bib53] Voulalas P.J., Schetz J., Undieh A.S. (2011). Differential subcellular distribution of rat brain dopamine receptors and subtype-specific redistribution induced by cocaine. Mol. Cell. Neurosci..

[bib54] Watanabe M., George S.R., Seeman P. (1985). Dependence of dopamine receptor conversion from agonist high- to low-affinity state on temperature and sodium ions. Biochem. Pharmacol..

[bib55] Yung K.K., Bolam J.P., Smith A.D., Hersch S.M., Ciliax B.J., Levey A.I. (1995). Immunocytochemical localization of D1 and D2 dopamine receptors in the basal ganglia of the rat: light and electron microscopy. Neuroscience.

