# Supplementary material for: The influence of different cellular environments on PET radioligand binding: An application to D2/3-dopamine receptor imaging
Source: Neuropharmacology. 2014 Oct;85(100):305–13. doi: 10.1016/j.neuropharm.2014.05.040 (PMC4109028; doi:10.1016/j.neuropharm.2014.05.040)
Supplement: Supplementary file 1 [file mmc1.docx]

**Supplementary Data**

***Supplementary Table 1***

K*_D_* , B*_max_* and *in vitro* BP values for each radioligand in the three physiological environments in pig striatum.

| **Radioligand and buffer** | **K*_D_* (nM)** | **B*_max_* (fmol/mg protein)** | **B*_max_* (pmol/g tissue)** | ***In vitro* BP** | ***% of* Extra *in vitro BP_p_*** |
| --- | --- | --- | --- | --- | --- |
| [^3^H]Raclopride |  |  |  |  |  |
| Extra | 5.8 ± 0.6 | 153 ± 26 | 7.5 ± 1.4 | 1.3 ± 0.2 | 100 |
| Intra | 7.7 ± 1.4 | 150  ± 33 | 6.9 ± 1.4 | 1.0 ± 0.2 | 75 |
| Endo | 41.8 ± 29.2^**^ | 42 ± 21 | 2.8 ± 1.0 | 0.3 ± 0.2^*^ | 21 |
| [^3^H]PhNO |  |  |  |  |  |
| Extra | 1.3 ± 0.4 | 61 ± 7 | 3.0 ± 0.2 | 3.0 ± 0.6 | 100 |
| Intra | 1.3 ± 0.3 | 91 ± 10 | 3.9 ± 0.3 | 3.6 ± 0.8 | 121 |
| Endo | 3.1 ± 0.5^*^ | 55 ± 15 | 3.5 ± 0.9 | 0.2 ± 0.04^*^ | 5 |
| [^3^H]Spiperone |  |  |  |  |  |
| Extra | 0.2 ± 0.1 | 145 ± 17 | 8.0 ± 1.6 | 57.8 ± 14.3 | 100 |
| Intra | 0.3 ± 0.1 | 186 ± 33 | 9.3 ± 2.1 | 53.1 ± 14.3 | 93 |
| Endo | 0.4 ± 0.2 | 127 ± 9 | 7.9 ± 0.8 | 35.6 ± 12.8 | 61 |

**Supplementary Data Legends**

***Supplementary Table 1***

K*_D_* (nM) and B*_max_* (fmol/mg protein and pmol/g tissue) and *in vitro* BP values for [^3^H]raclopride, [^3^H](+)PhNO and [^3^H]spiperone in Pig striatum (*n*= 4, mean ± s.e.mean) in the three ionic environments (Extra, Intra and Endo). One-way ANOVA with Tukey post-test were performed using SigmaStat 3.0. ^*^*p*< 0.05 and ^**^*p*< 0.01 represent comparison of intracellular or endosomal to the extracellular condition.

***Supplementary Figure 1***

Saturation curves performed with [^3^H]raclopride, [^3^H](+)PhNO and [^3^H]spiperone in Pig striatum (*n*= 4, mean ± s.e.mean) in the three ionic environments (Extra, Intra and Endo).
